# A Strip-Type Microthrottle Pump: Modeling, Design and Fabrication

**DOI:** 10.3390/s130303092

**Published:** 2013-03-04

**Authors:** Borut Pečar, Danilo Vrtačnik, Drago Resnik, Matej Možek, Uroš Aljančič, Tine Dolžan, Slavko Amon, Dejan Križaj

**Affiliations:** 1 Laboratory of Microsensor Structures and Electronics, Faculty of Electrical Engineering, University of Ljubljana, Tržaška 25, Ljubljana SI-1000, Slovenia; E-Mails: borut.pecar@fe.uni-lj.si (B.P.); danilo.vrtacnik@fe.uni-lj.si (D.V.); drago.resnik@fe.uni-lj.si (D.R.); matej.mozek@fe.uni-lj.si (M.M.); uros.aljancic@fe.uni-lj.si (U.A.); tine.dolzan@fe.uni-lj.si (T.D.); slavko.amon@fe.uni-lj.si (S.A.); 2 Centre of Excellence Namaste, Jamova 39, Ljubljana SI-1000, Slovenia; 3 Laboratory for Bioelectromagnetics, Faculty of Electrical Engineering, University of Ljubljana, Tržaška 25, Ljubljana SI-1000, Slovenia

**Keywords:** microthrottle pump, micropump, numerical simulation, 3D fully coupled model, hyperelastic model, PDMS, lab on chip, COMSOL, fluidics, optimization

## Abstract

A novel design for a strip-type microthrottle pump with a rectangular actuator geometry is proposed, with more efficient chip surface consumption compared to existing micropumps with circular actuators. Due to the complex structure and operation of the proposed device, determination of detailed structural parameters is essential. Therefore, we developed an advanced, fully coupled 3D electro-fluid-solid mechanics simulation model in COMSOL that includes fluid inertial effects and a hyperelastic model for PDMS and no-slip boundary condition in fluid-wall interface. Numerical simulation resulted in accurate virtual prototyping of the proposed device only after inclusion of all mentioned effects. Here, we provide analysis of device operation at various frequencies which describes the basic pumping effects, role of excitation amplitude and backpressure and provides optimization of critical design parameters such as optimal position and height of the microthrottles. Micropump prototypes were then fabricated and characterized. Measured characteristics proved expected micropump operation, achieving maximal flow-rate 0.43 mL·min^−1^ and maximal backpressure 12.4 kPa at 300 V excitation. Good agreement between simulation and measurements on fabricated devices confirmed the correctness of the developed simulation model.

## 1. Introduction

One of the main challenges in the development of micropumps appropriate for integration in complex lab-on-chip devices (LOC), total analysis systems (μTAS) and other MEMS [1,2], is to enable separate individual pumping of various liquids in parallel at efficient chip surface consumption. Here, various structures of piezoelectric actuator driven micropumps are described. In particular, we found that recently introduced piezoelectric microthrottle (MT) pumps provide good performance [3–6]. These devices are based on throttle valves which do not completely close during operation but rather exhibit sequential openings and closings gated by piezoelectric actuators. Absence of freely moving parts, in conjunction with the capacity for partial MT closing and simple design represent advantages, especially in the biomedical field where biological cells should not to be destroyed (squeezed) during pumping. Johnson *et al.* [3] proposed a MT pump based on a polydimethylsiloxane (PDMS) substrate with microvalves (throttles) and a glass membrane covered with piezoelectric (PZT) actuators. They used three PZT actuators and achieved pumping through appropriate sequential actuation of the PZTs. In 2005 they proposed a modification of a MT pump comprising a single PZT actuator that simplified device actuation [4,5]. Efficiency of MT pumps was further improved by inclusion of a polymer photoresist (PMMA) ring inside a PDMS to enhance displacement amplification in an elastomeric substrate [6].

Here, we propose a modified design of MT pump that differs from reported ones in the shape of the actuator (piezoelectric) and the membrane. We find that the rectangular shape of the actuator and the membrane used in our “strip-type MT pumps” is superior to circular actuators and square membranes [4–6]. These modifications make such structures particular suitable for applications in case of several parallel microchannels, enabling individual channel pumping at more efficient chip surface consumption.

Numerical device simulation using finite elements discretization has been used for modeling operation of the pump and optimization of crucial design parameters. We developed a fully coupled three dimensional time dependent electro-structural mechanic-fluidic numerical simulation model in which three differential equations are coupled and solved for seven unknowns: electric potential, three structural mechanics displacement components and three components of fluid flow velocities. No reports were found in the literature on 3D numerical models of micropumps with check valves (including throttle type) with PZT actuation. The few investigations of this type mainly cover diffuser type micropumps. Two dimensional (2D) numerical simulation models were presented by Cui *et al.* [7] and Al-Hourani *et al.* [8]. 3D simulation models are more difficult to build compared to 2D models as they require more computer resources and are more difficult to solve. Additional difficulties encountered in 3D simulations are in simulations of large structures with small details (for instance check valves) that require careful meshing and solving strategies. Fan *et al.* [9] were able to show that the pumping efficiency at actuating frequencies over 7.5 kHz depends not only on the actuating frequencies and maximum membrane deflection, but also on its shape. Yao *et al.* [10] have built a coupled 3D model of a diffuser type micropump. They reported an increased numerical complexity of the model at higher actuating frequencies. Similar models (on diffuser type micropumps) were reported also in references [11–13]; the simulation results of the last two references show good agreement with measurements on fabricated devices. An alternative to numerical modeling is to design microfluidic devices using electric circuit analogy, as recently reviewed by Oh *et al.* [14]. This approach however requires significant simplifications of the geometry as well as the physical phenomena.

Numerical simulation enabled detail analysis of device operation at a frequency of 1 Hz. Differences between the low (1 Hz) and high frequency (>10 Hz) operation are identified and related to the membrane deflection and the fluid inertia. We used numerical simulation to optimize two crucial design parameters of a proposed strip-type MT pump: height and position of the throttles. These data were used in design and fabrication of a prototype MT device. Measurements were performed on a developed device and the results are compared to the numerical simulation results.

## 2. Design and Fabrication of a Strip-Type Microthrottle Pump

**Figure 1.**
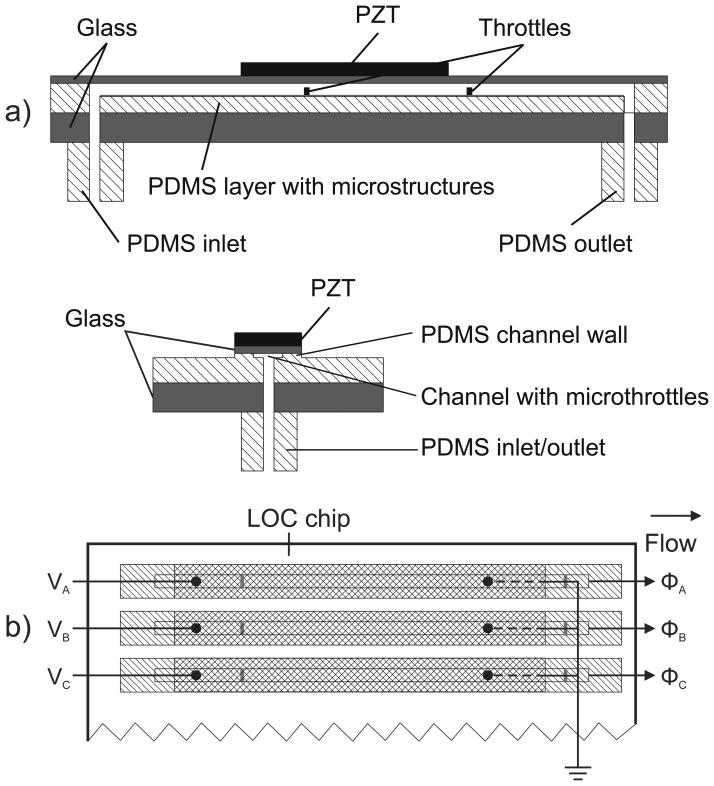
(**a**) Lateral and transversal cross-section of the proposed strip-type MT pump. (**b**) Three strip-type MT pumps compact connection for individual pumping.

Figure 1(a) presents lateral and transversal cross-section of the proposed MT pump with rectangular (strip-type) PZT actuator and Figure 1(b) an example of the compact parallel connections of three strip-type MT pumps for individual pumping at more efficient chip surface consumption as compared to standard micropumps with circular actuators.

**Figure 2.**
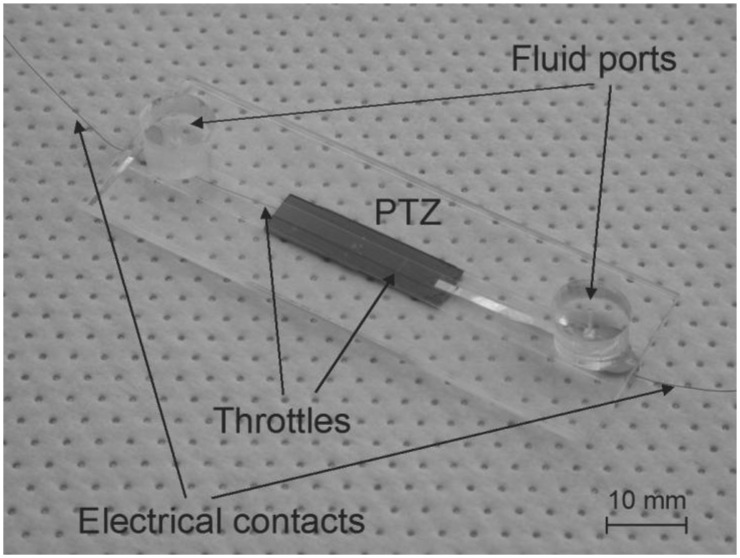
Fabricated strip-type MT pump.

The glass-PDMS-glass-PZT structure is in short described as follows: PDMS part of the micropump (cavity, fluid channel and throttles) was fabricated using soft lithography. A PDMS Sylagard^®^ 184 (Dow Corning Corporation) two-part kit consisting of a pre-polymer (base) and a cross-linker (curing agent) mixed at a ratio 10:1 was applied. A soft replica was fabricated from a silicon mold by using a two-step photolithography and Deep Reactive Ion Etching (DRIE) of silicon. Oxygen plasma activation was used to seal (bond) the PDMS layer between a thicker supporting bottom glass and a thin rectangular shaped glass membrane. Fluid ports were also made from PDMS and bonded onto the supporting bottom glass by oxygen plasma activation. Micropump is driven by a PZT actuator (PIC 255, PI Ceramic^TM^) that was glued on the top of the glass membrane. Figure 2 presents a fabricated strip-type MT pump.

## 3. Numerical Simulation Model

Numerical simulation software Comsol Multiphysics (Ver. 4.2a) has a special module for simulation of MEMS structures that includes piezoelectric materials [15]. In the following a brief description of relevant equations, boundary conditions, material parameters and a solution strategy is given.

Fluid flow is described by the Navier-Stokes equation:

(1) $$\rho \frac{\partial \nu }{\partial t}+\rho (\nu \cdot \nabla )\nu =\nabla \left[-p\text{I}+\mu \left(\nabla \nu +{\left(\nabla \nu \right)}^{T}\right)\right]+F$$

where the left hand side represents contribution of the force acting on a differential volume of a fluid and the inertial force. ***v*** is the fluid velocity, *ρ* density, *p* pressure and *μ* dynamic viscosity. The above equation describing conservation of momentum needs to be solved together with equation of mass continuity which for incompressible fluid reads:

(2) ∇⋅ν=0

Deformation of a structure due to movement of a piezoelectric actuator is modeled by structural mechanics equation for displacement vector ***u***:

(3) $$f_{u}=\rho \frac{\partial^{2}u}{\partial t^{2}}-\nabla \cdot \sigma$$

where *f_u_* is a force acting on a differential volume and *σ* is a stress tensor. Stress *σ* and strain *ε* tensors are related through equation ***σ*** = ***c****_E_****ε*** where ***c****_E_* is the elasticity tensor (determined with Young modulus of elasticity and Poisson ratio). In case of piezoelectric materials the stress also depends on the electric field *E* through electromechanical coupling tensor ***e: σ*** = ***c****_E_****ε*** − ***e****^T^****E***. PDMS is a rubber like material and is most appropriately described by a hyperelastic model. In this work a Mooney-Rivlin two parameters hyperelastic model based on strain energy density is used [16]. Also, when piezoelectrics are mechanically deformed they induce electric charge which is written as ***D*** = ***eε*** + *ε_0_****ε****_rS_****E*** where *D* is the electric displacement vector and ***ε****_rS_* is the relative permittivity tensor. Strain tensor is further related to displacement gradient through ***ε*** = ½ [(∇***u***)*^T^* + ∇***u***].

Electric field is given by a Gauss law:

(4) $$\nabla \cdot D=\rho_{e}$$

Where *ρ_e_* denotes the volume electric charge density (in our case equal to zero). A voltage driving signal is used to deform the piezoelectric therefore a relation ***E*** = −∇*V* is required with *V* being electric potential. Equations (1) to (4) are solved for fluid velocities, structural deformations and electric potential together with supporting relations described in the text.

**Figure 3.**
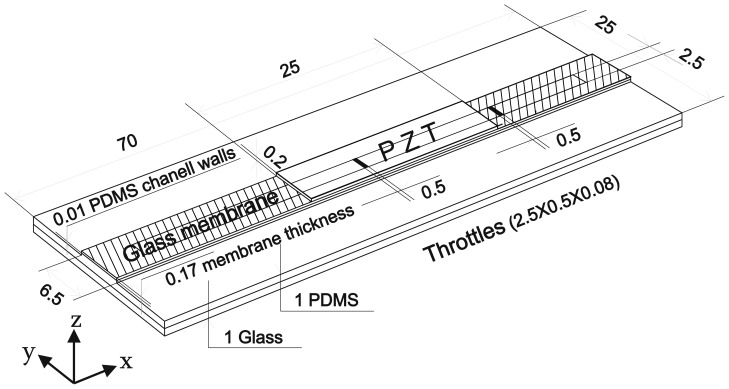
Geometry and dimensions of the analyzed structure (dimensions are not to scale).

Figure 3 presents the geometry of the analyzed structure. The total length and width of the simulated structure (also used in the design prototype) are 70 mm and 25 mm, respectively. The channel width and height are 2.5 mm and 0.1 mm, while the width and thickness of the glass membrane are 6.5 mm and 0.17 mm, respectively. The material properties for glass and water are taken from the program library of material properties, while for the PZT actuator the elasticity tensor ***c****_E_*, the electro-mechanical coupling tensor ***e***, density *ρ* and the relative permittivity tensor ***ε****_rs_* were constructed from the data provided by the PZT material supplier (PIC255, PI Ceramic^TM^). A two-component epoxy adhesive layer between the glass membrane and the PZT is considered to be very thin. As we were not able to measure its thickness its influence was not taken into account in the simulation model.

For PDMS material the density *ρ* = 968 kg·m^−3^ [17], Mooney-Rivlin model parameters *C*_01_ = 0.05 MPa, *C*_10_ = 0.25 MPa [18] and bulk modulus *K* = 1 GPa [17] were used. Several models for the fluid velocity at the channel walls exist in the literature [19–21]. Although the used software enables use of more complex models such as viscous slip and Maxwell's model [22], reliable parameter values for these models for water on PDMS surface are not available at the present time due to insufficient experimental measurements [23]. Therefore, in our simulation we modeled fluid velocity at the boundary with no-slip (zero fluid velocity at the boundary in all directions) or slip boundary condition (no viscous effects at the boundary resulting in zero fluid velocity only perpendicular to the boundary).

While the no-slip condition is physically more correct, the simulations using the slip condition are usually numerically easier to solve [24]. It will be shown that the difference between the no-slip and slip boundary condition are significant especially at higher frequencies (*f* > 70 Hz) of the excitation signals (larger flow-rates).

The complete mesh was constructed of 116,487 (tetrahedral, prism, pyramid, triangular) elements. Fluidic mesh was densified in the throttles region. Size of the elements in this region was decreased down to 3 μm, while maximum allowed elements size in the remaining channel was set to 200 μm. Usage of coarser mesh (52,430 elements) resulted in less accurate solution (up to 30% deviation as compared to the proposed mesh) for the outlet fluid flow and was therefore inappropriate for simulation purposes.

The solver can be set as a segregated or a fully coupled. In the first case the electro-mechanical solution is obtained first for structural deformations of the channel wall boundaries including the membrane, channel bottom and the throttles. Then the simulation model is solved for the fluid velocity and the pressure. The boundary pressure is fed back to the electro-mechanical solver as a boundary load ***F****_A_*=*p*·***n*** on all fluid channel wall boundaries including the membrane, channel bottom and the throttles, where ***n*** is a normal to the surface. This approach requires less computer memory space, but can lead to weak convergence, especially in cases of strong coupling between the variables. In our approach a fully coupled approach was used (all equations are solved simultaneously) leading to better convergence in particular at frequencies higher than 10 Hz where segregated solver was not able to converge. Another important step for achieving solver convergence was appropriate scaling of dependent variables in particular the fluid pressure *p*. Typical magnitude of the scaling factor for the pressure was 10^5^.

## 4. Numerical Simulation Model

### 4.1. Operation of the Strip-Type MT Pump-Numerical Modeling

**Figure 4.**
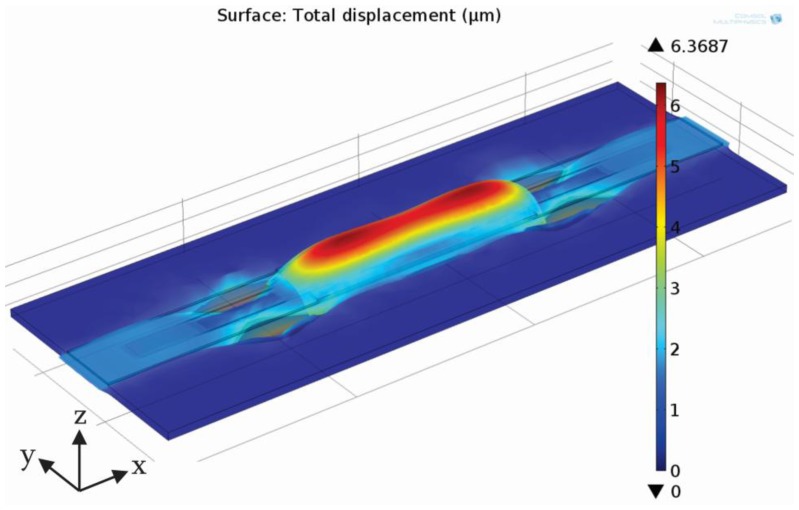
Deflection of the membrane together with a PZT actuator at maximal value of the excitation signal (300 V). Scaling factor in *z* direction is 500.

The piezoelectric was biased with a sinusoidal voltage signal of amplitude 300 V and a frequency of 1 Hz starting at time 0 s. Figure 4 presents maximal deflection of the membrane and the PZT occurring at maximal applied voltage (300 V). Maximal deflection is small (in the order of a few microns) so for visualization purposes membrane deflection is scaled up 500 times. In the region of a PZT the membrane deflects in the positive *z* direction. A saddle shape is a result of a magnitude of an applied signal and a length of a PZT and is typical for longer strip-type MT pumps (it cannot be observed in disk-type PZT actuators). On the other hand, the membrane deflects in a negative *z* axis direction away from the PZT edge. Four deflection maxima are observed at the corners of the PZT, which are a consequence of a rectangular shape of the PZT and increased electric field at the corners. Simultaneous membrane deflection in positive and negative *z* axis is a key element that enables sequential opening and closing of the throttles during device operation. For proper device operation one of the throttles is positioned in the region of maximal positive membrane deflection, while the other one on the opposite side in the region of maximal negative membrane deflection. The shape of the membrane deflection therefore strongly influences device operation and optimization of design parameters—in particular in proper positioning of the throttles.

**Figure 5.**
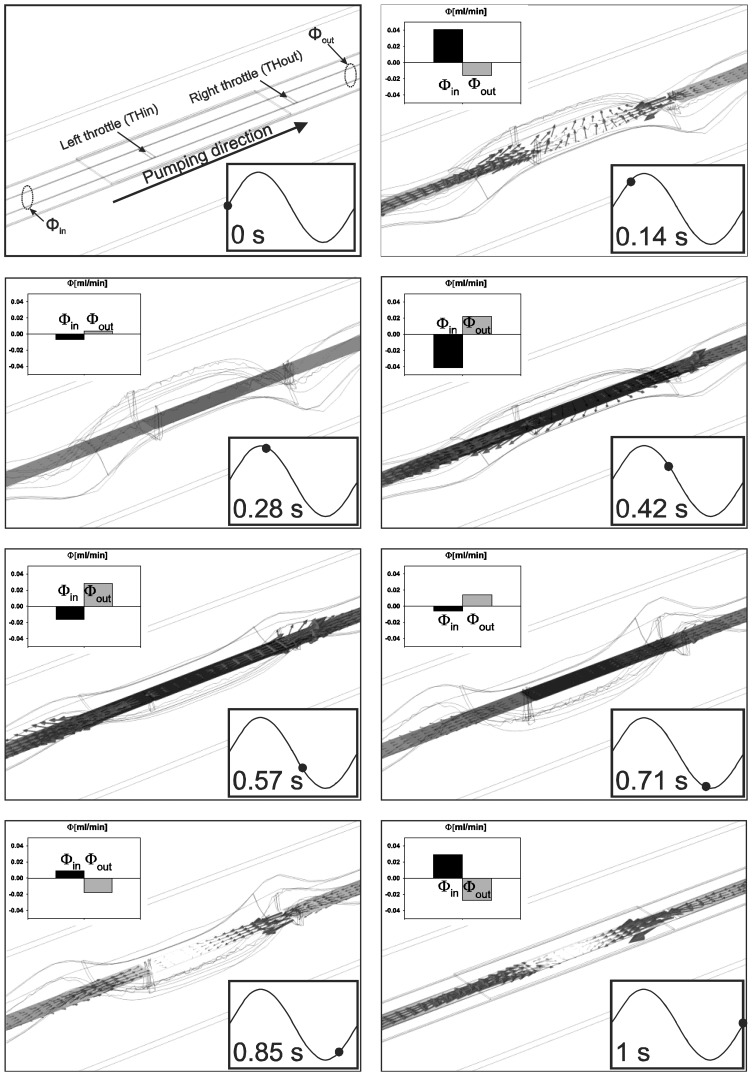
Structural deformations, pressure distribution and fluid velocity (vectors) for selected time frames (1 Hz, 300 V).

Figure 5 presents operation details through 3D graphs of structural deformations, pressure distributions and vectors of fluid velocity of the simulated strip-type MT pump for selected time frames considering a sinusoidal excitation signal frequency of 1 Hz and amplitude of 300 V (see also supplementary file with video animation of deformation, pressure and velocity). Inside each frame a volume flow-rate at inlet and outlet at specified times is presented as well. Volume flow-rate is computed as a surface integral of a normal fluid velocity at selected cross-sections and expressed in mL·min^−1^. For positive applied excitation signal the middle part of the membrane (together with a PZT) moves upwards while the part of the membrane not covered by the PZT moves downwards. This enables widening (opening) of the channel cross-section at the left throttle and narrowing (closing) of the channel cross-section at the right throttle. This can be regarded as a suction phase. During the suction phase the volume between the throttles is increased, however, the flow through the outlet is negative (time frame 0.14 s, Φ_out_ < 0) meaning that the fluid flows into the pump also from the outlet. At maximal excitation signal the membrane is maximally deflected. In this particular moment there is no volume change and due to a low frequency of the applied signal there is also no fluid inertia. Consequently, the volume flow-rate is zero at this moment. During the second quarter of the period (time frame 0.28 s and 0.42 s) the membrane deflection is reducing, the left throttle closes and the right throttle opens. During this-pumping-phase, the volume between the throttles decreases, resulting in a fluid flow in both directions towards the inlet and the outlet. In fact, more fluid flow is observed towards the inlet than the outlet.

The pumping phase continues also through the third quarter of the period when the membrane deflects in the negative *z* axis (toward the bottom of the channel) and further presses the fluid towards the outlet (time frame 0.57 s and 0.71 s). This is a consequence of further closure of the left throttle and opening of the right throttle. Also during this time period the flow is observed in both directions, however, it is larger towards the outlet than the inlet. During the fourth quarter of the period the membrane deflection reduces resulting in a repetition of the suction phase. During this time the negative fluid flow-rate at outlet is observed once again (time frame 0.85 s).

**Figure 6.**
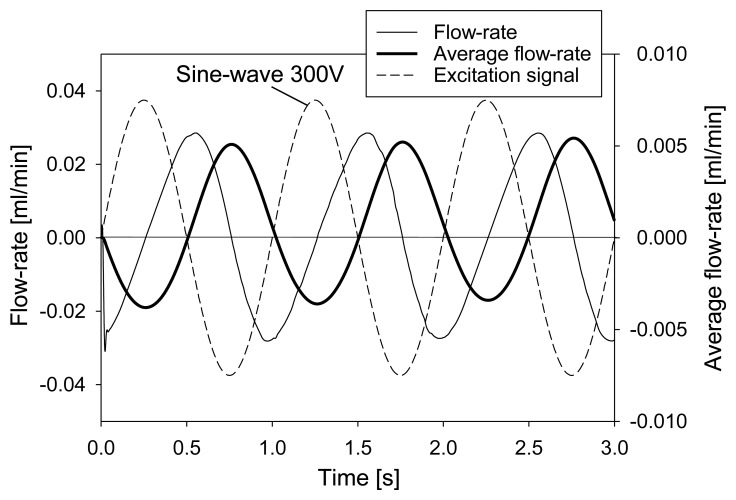
Numerical simulation of time dependent fluid flow at the channel outlet as a consequence of 1 Hz 300 V sinusoidal excitation signal.

Average flow-rate at the outlet can be computed from simulation results by time integration of the outlet flow-rate divided by the integration time. These results are presented together with the applied excitation signal and the flow-rate in Figure 6. As already explained, the flow-rate is negative during the first and the fourth quarter and positive during the second and the third quarter of the period. A time lag (phase shift) of about a quarter of a period between the excitation signal and the net flow-rate at the outlet can be observed. This result is expected, as the membrane deflection is in phase with the excitation signal, while the flow-rate is zero during maximal membrane deflection. Furthermore, the flow-rate is maximal during times of maximal change of membrane deflection which occurs when the excitation signal changes polarity. Time averaged flow-rate results in net pumped volume, which is for 1 Hz/300 V excitation slightly positive after one period indicating proper device operation but with poor performance (about 0.0015 mL·min^−1^).

**Figure 7.**
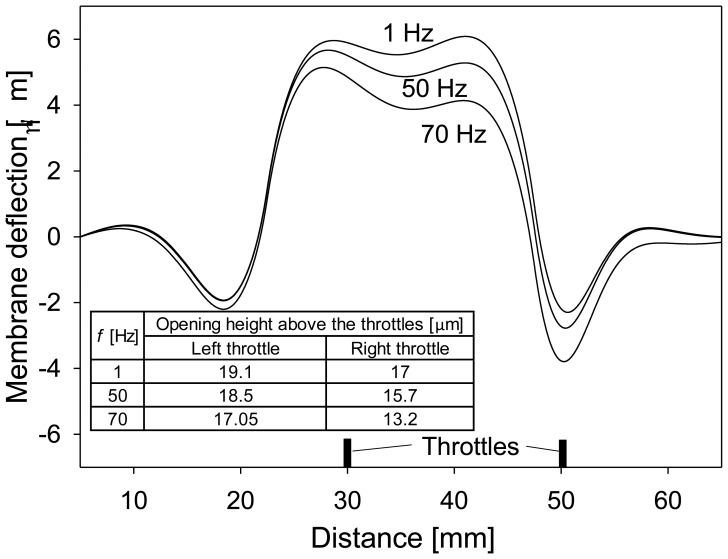
Numerical simulation of membrane deflection at maximal excitation signal (300 V) in a middle cross-section along the channel for three frequencies: 1 Hz, 50 Hz and 70 Hz. An insert provides data on of the channel opening height.

The pump operation at higher frequencies (10 Hz–70 Hz) of the applied excitation signal differs substantially from the low frequency operation (1 Hz) due to differences in the speed and shape of the membrane deflection, which result in different maximal volumes between the throttles and opening heights at the throttles. Figure 7 presents deflection of the membrane in the middle cross-section along the channel at maximal excitation signal (300 V) for three frequencies of the applied signal: 1 Hz, 50 Hz and 70 Hz. Membrane deflection is reduced at higher frequencies leading to reduced maximal volume between the throttles. This could result in less efficient pumping, however, at the same time the opening height above the throttles is reduced which results in more efficient closure of the throttles and thus more efficient pumping. Figure 7 also indicates that the position of maximal negative and positive deflection depends on the frequency of the applied signal (and also on the magnitude of the applied signal, but this is not shown in the figure). This is important as these positions can be used to determine optimal positions of the throttles.

**Figure 8.**
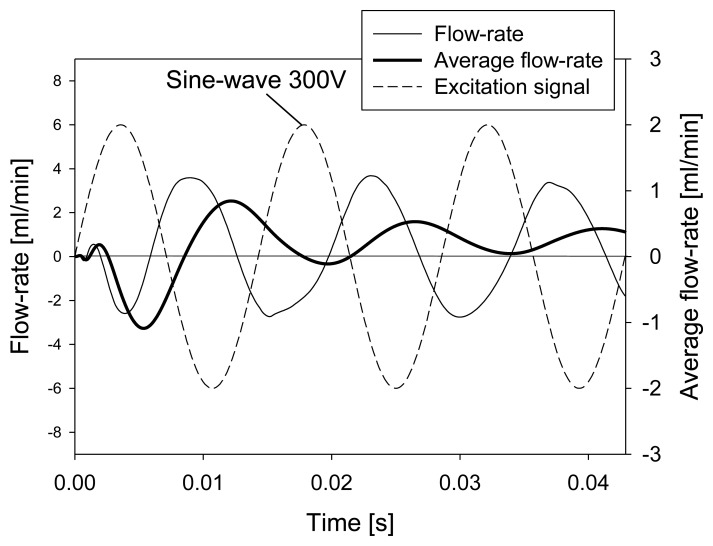
Numerical simulation of time dependent fluid flow at the channel outlet as a consequence of 70 Hz 300 V sinusoidal excitation signal.

Figure 8 presents the same variables as in Figure 6, but for a frequency of 70 Hz. In contrast to operation at 1 Hz, the negative outlet flow-rate is delayed and reduced. This can be explained by the fact that the membrane at the right throttle moves downwards very fast and closes the channel at the throttle more efficiently than at 1 Hz, pushing the fluid in both directions and thus also through the outlet. A larger phase shift between the excitation signal and the flow-rate at outlet is a consequence of an inertial force that increases at increased frequencies of the excitation signal. Besides, the closure of the throttles is much more efficient at 70 Hz resulting in less negative outlet flow-rate and consequently in increased average flow-rate. It should be noted that the scales in Figures 6 and 8 for average flow-rate differ by more than two decades, indicating that at increased frequency of the excitation signal the average flow-rate is increased. This is presented in more detail in Subsection 4.3 where average flow-rate obtained by numerical simulation is compared to experimental results.

### 4.2. Determination of Optimal Positions and Height of the Throttles by Numerical Simulation

**Figure 9.**
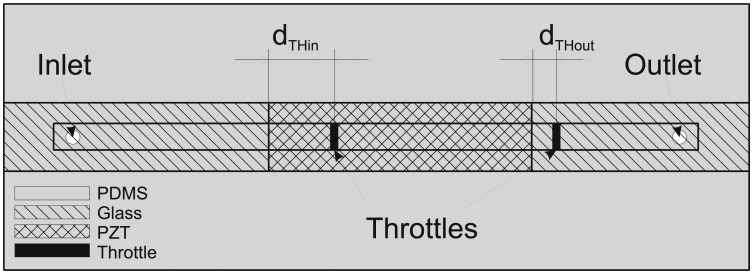
Design details of the MT pump and positioning of the throttles.

The main goal of optimization was to determine the positions of throttles that would result in maximal average flow-rate. As already discussed in Subsection 4.1, optimal throttle positions and heights depend on several parameters in particular on the frequency and amplitude of the excitation signal. Measurements as well as simulation (see Figure 12 below) have shown maximal performance (average flow-rate) between 60 and 80 Hz. Therefore optimization was performed at a frequency of 70 Hz and 300 V amplitude of the excitation signal. From this section on, the average flow-rates were determined from simulation results after five periods of the excitation signal. The positions determined by this procedure were used in design and fabrication of a prototype strip-type micropump. According to Figure 9, we define positions of the throttles by the distances from the left and the right edge of the PZT (d_THin_ and d_THout_, respectively).

**Figure 10.**
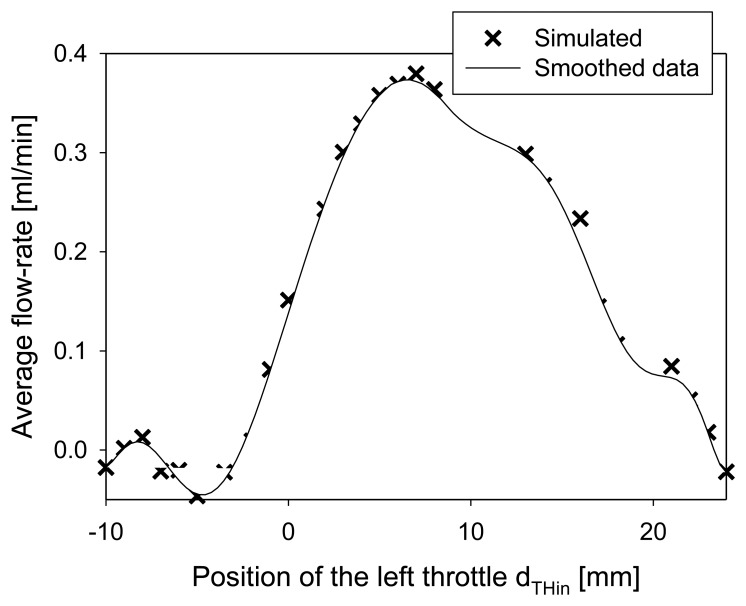
Numerical simulation of average flow-rate depending on the position of the left throttle after five time periods (70 Hz, 300 V). Right throttle is fixed at 3 mm.

The optimal position of the right throttle was determined from the position of maximal negative membrane deflection which occurred at d_THout_ = 3 mm. The optimal position of the left throttle has been obtained from a study of average flow-rates at varying positions of the left throttle. As shown in Figure 10, maximal average flow-rate was obtained at d_Thin_ = 8 mm, which was thus chosen as a position of the left throttle in the design of a prototype device.

**Figure 11.**
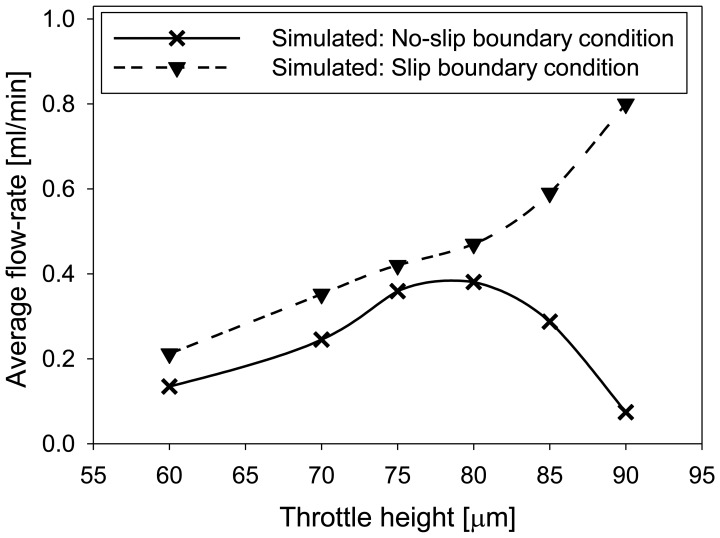
Numerical simulation of average outlet flow-rate for varying throttle heights after five time periods (70Hz, 300V).

Influence of throttle height on average flow-rate was analyzed at optimized positions of the throttles determined in the previous paragraph for two different boundary conditions for the fluid velocity (Figure 11). For slip boundary condition the average flow-rate was increased at larger throttle heights. More realistic results were obtained for no-slip boundary condition resulting in maximum of average flow-rate at a throttle height of 80 μm (the total channel height was 100 μm) followed by a decrease for larger throttle heights. This decrease is a consequence of a very small opening at the throttles which due to zero fluid velocity at the boundaries (no slip boundary condition) additionally decreases the fluid flow.

### 4.3. Experimental Results and Comparison with Numerical Simulation

Strip-type MT pump prototype was designed according to the processing steps in Section 2 and device dimensions described in Section 3 and the positions and heights of the throttles determined through numerical simulation (Section 4). Flow rates were measured on a prototype device using a volumetric method while the back pressure was determined by measuring the height of the fluid column. DI water was used as a pumping fluid in all measurements.

**Figure 12.**
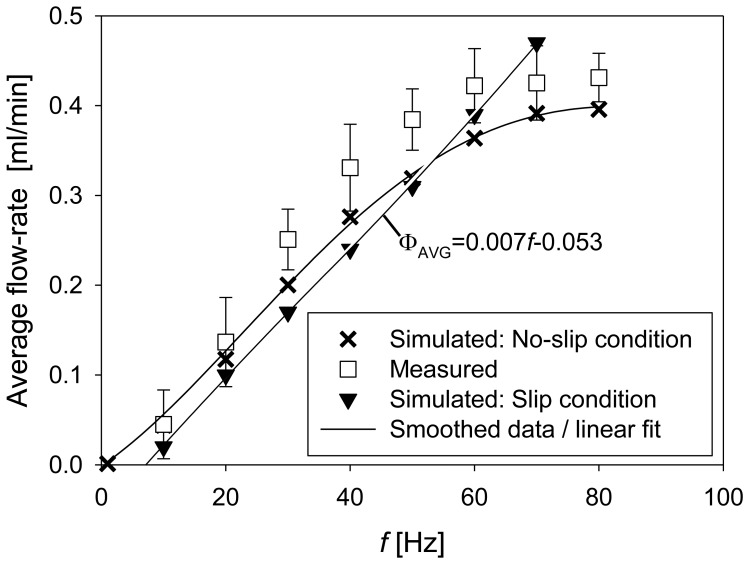
Average flow-rate depending on the frequency of the applied excitation signal (at amplitude of 300 V).

The average flow-rate is increased with increasing frequency of the excitation signal, as presented in Figure 12. Error bars represent the standard deviation derived from five measurements. Average flow-rates on a fabricated prototype increased approximately exponentially but can also be described as linear for frequencies below 50 Hz (at a rate of 0.0087 mL·min^−1^·Hz^−1^) saturating to a value of about 0.43 mL·min^−1^ at a frequency of 60 Hz. Numerical simulation results are shown for two different boundary conditions for the fluid flow at the channel walls. For the slip boundary condition a linear increase with frequency at a rate of 0.007 mL·min^−1^·Hz^−1^ was obtained and no saturation effect is observed. More realistic results were obtained for no-slip boundary condition resulting in a similar shape of average flow-rate increase but on average about 0.03 mL·min^−1^ smaller than experimentally determined. Average flow-rate saturated at a frequency of 70 Hz. The differences between the measurements and the simulation can be a consequence of several elements starting from non-ideal fabrication process to non-suitably chosen material parameters. In particular, the material parameters of the PDMS can vary substantially depending on the processing steps in particular on the curing temperature and the proportion of the curing agent [25]. Further increase of the excitation frequency may result in several additional effects that could influence device operation such as turbulent flow around the throttles, excessive heating of the fluid resulting in changes of the fluid viscosity or even fluid cavitation due to rapid changes in the pressure. The model used in the study does not cover these effects. Solver convergence was not problematic at lower frequencies (1 Hz) while at higher frequencies (above 10 Hz) additional solver tuning was necessary. At frequencies higher than 80 Hz convergence problems were encountered. The measurements were nevertheless performed up to a frequency of 500 Hz without a noticeable increase of the average flow-rate.

**Figure 13.**
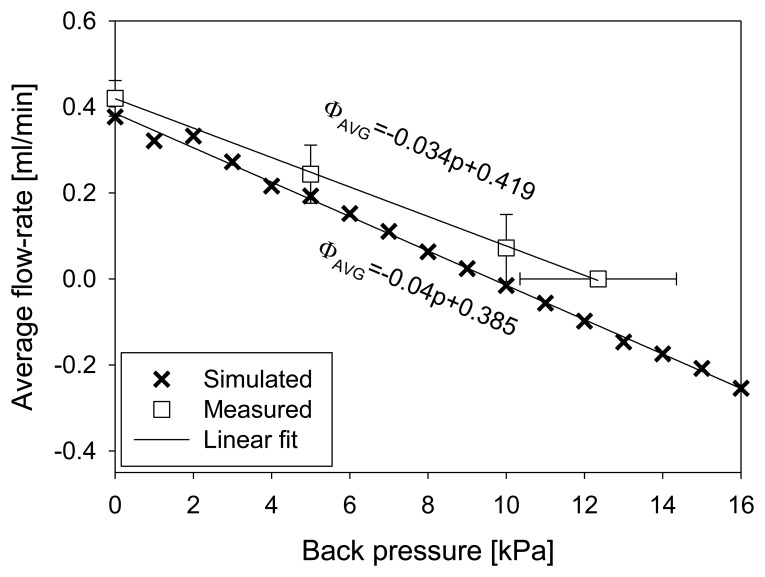
Average flow-rate depending on the back pressure (70 Hz, 300 V).

The throttles never completely close the channel during operation. For this reason they are additionally susceptible to the increased back pressure. Figure 13 shows experimental and simulation results of average flow rate dependency on backpressure, obtained at a frequency of 70 Hz and 300 V excitation signal. Error bars represent the standard deviation derived from five measurements. For simulation purposes, back pressure was applied on micropump outlet boundary as a pressure boundary condition. A linear decrease of average flow-rate was obtained in both cases. Experimentally determined rate of decrease was 0.034 mL·min^−1^·kPa^−1^ reaching zero at a back pressure of 12.4 kPa. The point of zero average flow was determined by the maximal height of the fluid column (DI water). Numerically obtained rate of decrease was 0.04 mL·min^−1^·kPa^−1^ reaching zero at a back pressure of 9.5 kPa.

**Figure 14.**
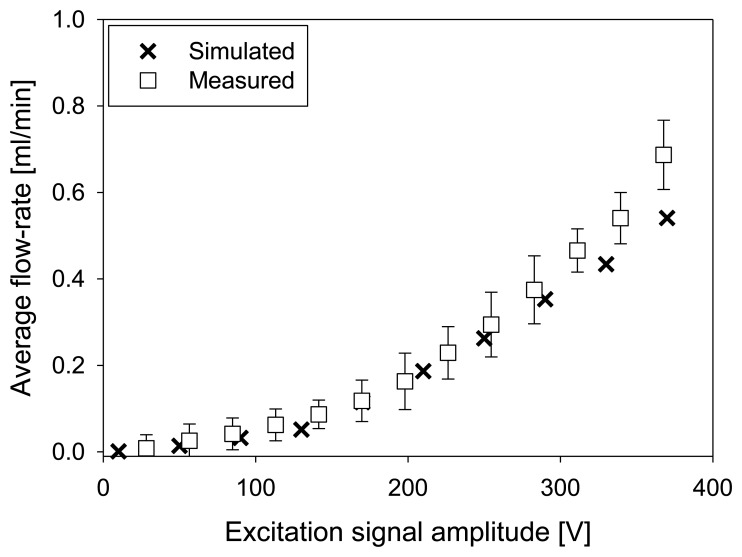
Average flow-rate depending on the amplitude of the excitation signal (at frequency of 70 Hz).

Increased amplitude of the excitation signal increases also the magnitude of the membrane deflection. Here, it should be noted that membrane deflection depends also on the frequency of the applied signal as presented in Figure 7. Figure 14 presents experimental and numerical simulation results of average flow-rate at a frequency of 70 Hz. Error bars represent the standard deviation derived from five measurements. A good agreement between experiment and numerical simulation expressing approximately quadratic increase of average flow-rate has been obtained up to amplitude of excitation signal of 300 V. Further increase is in practice limited by the PZT depolarization [26] and/or by irreversible actuator damage at very large membrane deformations [27].

## 5. Conclusions

In the present work a novel, rectangular strip-type micro-throttle pump is proposed and investigated by numerical device simulation as well as experimental work. The pump structure is based on rectangular actuator geometry, resulting in more efficient chip surface consumption compared to existing micropumps with circular actuators. Proper determination of design parameters was made possible by development and usage of a fully coupled electro-fluid-structural mechanics 3D numerical simulation model in COMSOL Multiphysics. Only after taking into account the inertial fluid flow, no-slip boundary conditions and hyperelastic material model for the PDMS, did simulations yield good agreement with measured results. In order to assure convergence and accuracy of the simulation results the mesh has been locally refined, in particular in the regions near the throttles. Convergence has been improved after the variables, in particular the pressure, were appropriately scaled. Significant differences in simulation results were obtained for boundary conditions taking into account zero-fluid velocity at the channel walls (no-slip condition) and non-zero boundary fluid velocity (slip condition). More accurate results were obtained with the no-slip condition.

Numerical simulations were used for optimization of important design parameters such as optimization of the position and height of the throttles. For optimal pump operation with sinusoidal actuation signal of amplitude 300 V and frequency 70 Hz, the throttles position were 8 mm and 3 mm from the PZT edge, for the right and left throttle, respectively. Optimal height of the throttles for a 100 μm channel height was determined at 80 μm.

The analyzed strip-type micropump structure was designed and fabricated in accordance to the optimal positions and height of the throttles found by numerical simulation. Measurements on fabricated prototypes revealed saturation of average flow-rate to a value of 0.43 mL·min^−1^ at a frequency of 60 Hz. In simulations, the average flow-rate saturated to a value of 0.39 mL·min^−1^ at a frequency of 70 Hz. The agreement between simulated and measured results was particularly good at low (10 Hz) and higher (above 50 Hz) frequencies. Average measured flow-rate linearly decreases with back-pressure at a rate of 0.034 mL·min^−1^·Pa^−1^, reaching zero at a back-pressure of 12.4 kPa. Numerically obtained rate of decrease was 0.04 ml·min^−1^·Pa^−1^ reaching zero at a back pressure of 9.5 kPa. Good agreement between measurements and simulations was found for average flow rate increase with increasing amplitude of the excitation signal in particular up to 300 V. Approximately quadratic increase was obtained in both cases.

The agreement between simulation and measurements on fabricated devices confirmed the correctness of the developed simulation model. Measured pump characteristics proved expected micropump operation, achieving maximal flow-rate 0.43 mL·min^−1^ and maximal backpressure 12.4 kPa at 300 V excitation. Thus, the microthrottle simulation model proposed here might be useful in aiding further optimizations of the device as well as facilitate the design of improved micropumps based on piezoelectric actuation.

## Supplementary Files

Supplementary File 1 AVI-Document (AVI, 5046 KB)
